# A Case of Atraumatic Acute Carpal Tunnel Syndrome Requiring Emergency Surgery

**DOI:** 10.7759/cureus.44935

**Published:** 2023-09-09

**Authors:** Kentaro Ohuchi, Tatsuru Tomioka, Naohisa Miyakoshi

**Affiliations:** 1 Orthopedic Surgery, Yokote Municipal Hospital, Yokote, JPN; 2 Orthopedic Surgery, Akita University Graduate School of Medicine, Akita, JPN

**Keywords:** acute inflammation, synovectomy, emergency surgery, carpal tunnel pressure, acute carpal tunnel syndrome

## Abstract

Acute carpal tunnel syndrome (ACTS) is an urgent condition in which symptoms progress rapidly on an hourly basis, and emergency surgery may be required to treat it. ACTS often occurs after a traumatic event such as a fracture of the distal radius, and rarely occurs non-traumatically. We present a case of a 60-year-old male with ACTS secondary to acute synovitis due to rheumatoid arthritis. The patient complained of strong numbness from the thumb to the ring finger and pain in the palm, and he was unable to actively flex or extend his fingers. In addition, severe tenderness was observed in the palm; on the contralateral side, no obvious tenderness of the forearm and wrist joint was observed. Due to the intolerable pain and numbness, ACTS was suspected. Internal pressure from the forearm to the palm was measured, and it was found that the internal pressure of the carpal tunnel was elevated at 150 mmHg. Based on these findings, non-traumatic ACTS was diagnosed, and emergency surgery was performed. The transverse carpal ligament was exposed, an incision was made from the distal end, and the proximal part was fully incised to the forearm fascia so that the carpal tunnel was completely released. The synovial membranes around the median nerve were peeled off, confirming that the nerve had been loosened sufficiently. After the operation, finger pain and numbness improved dramatically from the day after surgery. Proper diagnosis and prompt treatment with surgical median nerve decompression are crucial for good functional recovery in these patients.

## Introduction

Carpal tunnel syndrome (CTS) develops when the pressure in the carpal tunnel rises, and the median nerve is compressed [1]. The symptoms often progress slowly and chronically. On the other hand, acute carpal tunnel syndrome (ACTS) is an urgent condition in which symptoms progress rapidly on an hourly basis, and emergency surgery may be required. ACTS often occurs after trauma such as a fracture of the distal radius, and non-traumatic cases are rare [2]. In this report, we present a case of ACTS secondary to acute synovitis due to rheumatoid arthritis.

## Case presentation

**Figure 1:**
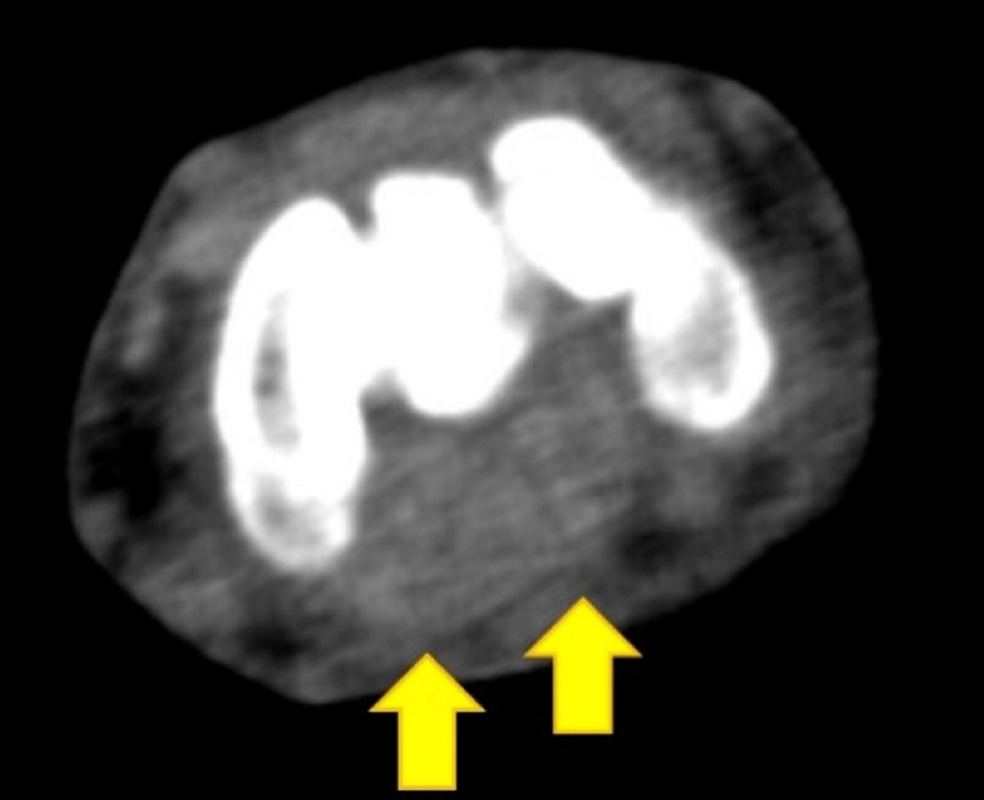
Contrast-enhanced CT of the patient The image showed a low-density area spreading around the flexor tendon, suggesting synovial hyperplasia, from the distal radioulnar joint to the carpal bone level CT: computed tomography

A 60-year-old male who was employed in the delivery industry where he mainly performed intense manual work or transportation of heavy objects visited the emergency outpatient department due to left wrist joint pain and numbness in the fingers that had started the night before. He had worked as usual the morning of the next day, but since the symptoms had gradually worsened, he presented to the emergency outpatient department. He had had swelling and pain in the proximal interphalangeal joint of both middle fingers for seven years and had received medication for the diagnosis of rheumatoid arthritis. The patient complained of strong numbness from the thumb to the ring finger and pain in the palm. Slight swelling of the fingers was observed, but no abnormalities in skin tone or skin temperature were noticed. He was unable to actively flex or extend his fingers, and passive extension of the fingers caused intense pain from the distal wrist to the palm. In addition, severe tenderness was observed in the palm; on the contralateral side, no obvious tenderness of the forearm and wrist joint was observed. No abnormal findings were found on the X-ray of the wrist joint. Contrast-enhanced CT showed a low-density area spreading around the flexor tendon, suggesting synovial hyperplasia, from the distal radioulnar joint to the carpal bone level (Figure 1).

Due to the intolerable pain and numbness, acute forearm compartment syndrome was suspected, and internal pressure from the forearm to the palm was measured. We set up a monitor and an arterial pressure measurement line, connected a 23-gauge needle locally, and inserted it to measure the internal pressure in real-time. It was found that the internal pressure of the forearm compartment was not significantly increased, but the internal pressure of the carpal tunnel was elevated at 150 mmHg. Based on these findings, non-traumatic ACTS was diagnosed, and emergency surgery was performed.

**Figure 2:**
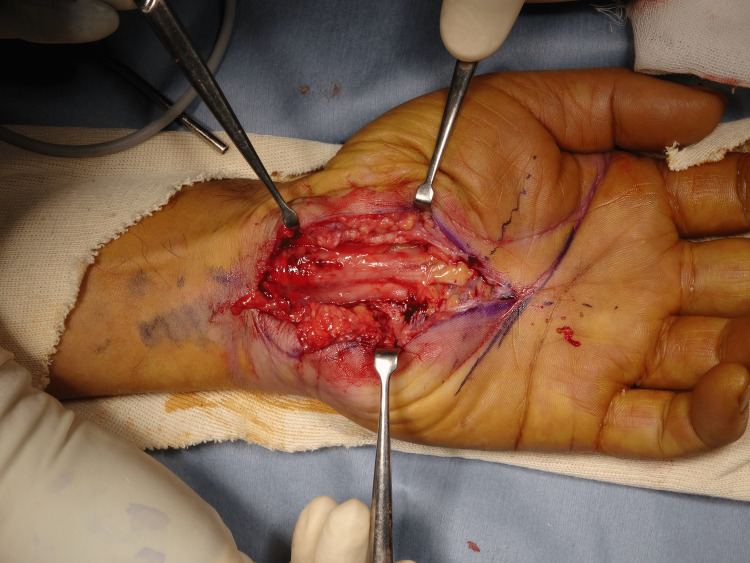
Intraoperative image 1 The median nerve was extensively discolored (red and swollen). In addition, synovial membranes had grown and adhered around the median nerve and flexor tendons

**Figure 3:**
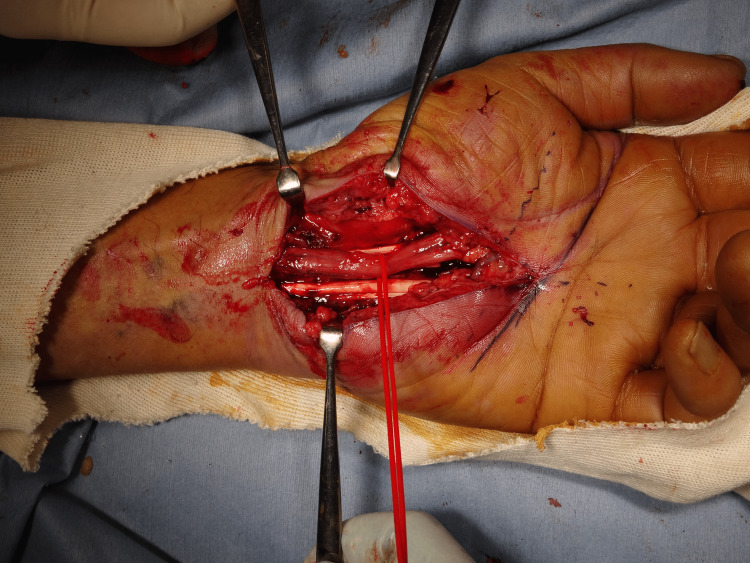
Intraoperative image 2 The synovial membranes around the median nerve were peeled off, confirming that the nerve had been loosened sufficiently. In addition, a synovectomy was performed until the flexor tendon was sufficiently slippery

**Figure 4:**
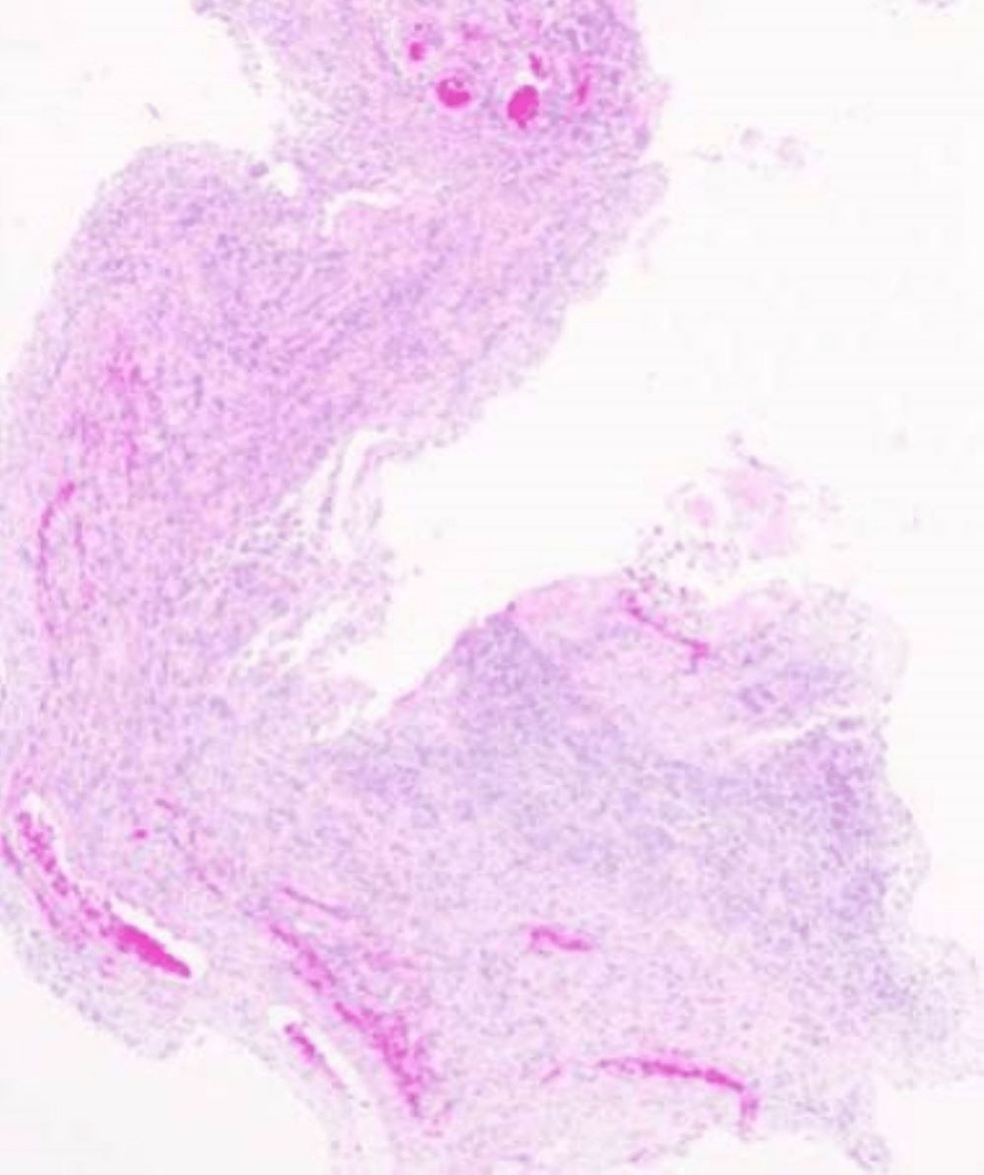
Pathological examination The resected synovium showed neutrophil infiltration and bleeding, findings suggestive of acute inflammation in chronic synovitis

Surgery was performed with a brachial plexus block through the axillary approach with a tourniquet. A skin incision was made from the distal end of the carpal tunnel to the proximal of the wrist skin line. The transverse carpal ligament was exposed, an incision was made from the distal end, and the proximal part was fully incised to the forearm fascia so that the carpal tunnel was completely released. The median nerve was extensively discolored and was found to be red and swollen. In addition, synovial membranes had grown and adhered around the median nerve and flexor tendons (Figure 2), and hence tendon gliding was strongly restricted. The synovial membranes around the median nerve were peeled off, confirming that the nerve had been loosened sufficiently. In addition, a synovectomy was performed until the flexor tendon was sufficiently slippery (Figure 3). On pathological examination, the resected synovium showed neutrophil infiltration and bleeding, findings suggestive of acute inflammation in chronic synovitis (Figure 4). 

After the operation, rehabilitation with the aid of an occupational therapist was started. The patient's finger pain started to improve dramatically from the day after surgery. One year after the surgery, the numbness of the fingers had disappeared; the range of motion of the fingers and grip strength had returned to pre-surgical levels, and he was back at the job he had engaged in before the surgery.

## Discussion

ACTS is a disease in which the pressure inside the carpal tunnel rises sharply for some reason, and the median nerve strangulation symptoms progress rapidly, peaking within a few hours. ACTS is often caused by trauma, such as distal radius fracture and carpal fracture, and non-traumatic onset is relatively rare [2]. Infection, bleeding, amyloidosis, and radiation have been cited as causes of non-traumatic onset [3-7]. In our case, the patient had chronic synovitis due to rheumatoid arthritis; external physical force was applied by the hand to induce acute inflammation, and the pressure inside the carpal tunnel rose sharply, causing ACTS.

For the diagnosis of ACTS, it is important to accurately evaluate the strangulation symptoms of the median nerve. Typical symptoms include severe numbness and pain in the thumb, index finger, middle finger, and radial side of the ring finger; however, the symptoms are often so severe that it is challenging to accurately evaluate the physical findings. In such cases, measurement of carpal tunnel pressure is effective as an objective evaluation method. The examination can be performed quickly with only a simple tool, and it is also useful for distinguishing the condition from acute forearm compartment syndrome by examining the pressure difference with the surrounding tissue. However, it has been observed that if a diagnosis of ACTS can be easily assumed based on clinical symptoms, it is unnecessary to spend time measuring the carpal tunnel pressure [8]; hence, it is important to make a judgment on a case-by-case basis.

Treatment of ACTS requires rapid decompression of the median nerve by surgery. There is no clear consensus on the timing of surgery, but the option of emergency surgery should be considered since its delay increases the risk of irreversible sequelae over time [9].

The surgical procedure for ACTS requires the complete release of the lateral carpal ligament. Stannard states that it is important to make a skin incision about 2 cm proximal to the wrist skin line to obtain a sufficient surgical field. In addition, it is necessary to prevent the carpal tunnel pressure from rising again by removing the tissue that has grown in the carpal tunnel [10,11]. In our case, a wide skin incision was made to the proximal wrist joint, and a sufficient incision was made to the fascia of the forearm to decompress the nerve and remove the enlarged synovium as much as possible. A good prognosis could be obtained without any residual disability.

## Conclusions

While ACTS is less common than chronic CTS, it is a disease that requires urgent care and should not be overlooked. ACTS often occurs traumatically, but it rarely occurs in the setting of minor physical activity. In our case, carpal tunnel pressure measurement proved to be useful for diagnosing ACTS. Proper diagnosis and prompt surgical median nerve decompression are important for good functional recovery.
